# Deep learning model for multi-classification of infectious diseases from unstructured electronic medical records

**DOI:** 10.1186/s12911-022-01776-y

**Published:** 2022-02-16

**Authors:** Mengying Wang, Zhenhao Wei, Mo Jia, Lianzhong Chen, Hong Ji

**Affiliations:** 1 Information Management and Big Data Center, Peking University Third Hospital, Beijing, China; 2 Goodwill Hessian Health Technology Co. Ltd, Beijing, China

**Keywords:** Infectious diseases, Multi-classification, Early diagnosis, Deep learning

## Abstract

**Purpose:**

Predictively diagnosing infectious diseases helps in providing better treatment and enhances the prevention and control of such diseases. This study uses actual data from a hospital. A multiple infectious disease diagnostic model (MIDDM) is designed for conducting multi-classification of infectious diseases so as to assist in clinical infectious-disease decision-making.

**Methods:**

Based on actual hospital medical records of infectious diseases from December 2012 to December 2020, a deep learning model for multi-classification research on infectious diseases is constructed. The data includes 20,620 cases covering seven types of infectious diseases, including outpatients and inpatients, of which training data accounted for 80%, i.e., 16,496 cases, and test data accounted for 20%, i.e., 4124 cases. Through the auto-encoder, data normalization and sparse data densification processing are carried out to improve the model training effect. A residual network and attention mechanism are introduced into the MIDDM model to improve the performance of the model.

**Result:**

MIDDM achieved improved prediction results in diagnosing seven kinds of infectious diseases. In the case of similar disease diagnosis characteristics and similar interference factors, the prediction accuracy of disease classification with more sample data is significantly higher than the prediction accuracy of disease classification with fewer sample data. For instance, the training data for viral hepatitis, influenza, and hand foot and mouth disease were 2954, 3924, and 3015 respectively and the corresponding test accuracy rates were 99.86%, 98.47%, and 97.31%. There is less training data for syphilis, infectious diarrhea, and measles, i.e., 1208, 575, and 190 respectively and the corresponding test accuracy rates were noticeably lower, i.e., 83.03%, 87.30%, and42.11%. We also compared the MIDDM model with the models used in other studies. Using the same input data, taking viral hepatitis as an example, the accuracy of MIDDM is 99.44%, which is significantly higher than that of XGBoost (96.19%), Decision tree (90.13%), Bayesian method (85.19%), and logistic regression (91.26%). Other diseases were also significantly better predicted by MIDDM than by these three models.

**Conclusion:**

The application of the MIDDM model to multi-class diagnosis and prediction of infectious diseases can improve the accuracy of infectious-disease diagnosis. However, these results need to be further confirmed via clinical randomized controlled trials.

## Background

Infectious diseases have accompanied human development at every stage and seriously threaten human health even today. Despite advances in medicine, infectious diseases are still the main cause of death, disease, disability, and socio-economic turmoil worldwide [1]. Early and correct diagnosis and the correct choice of treatment can considerably affect the outcome of any infection. China implements classified management of infectious diseases. The current statutory reported infectious diseases are divided into three categories: class A, B, and C. With COVID-19 being newly added in 2020, there are as many as 40 kinds of infectious diseases. The National Health Commission has decided to include certain infectious diseases under Class B and Class C infectious diseases for management, while other infectious diseases that are subject to emergency monitoring reports fall under Class A management [2]. Different management methods are adopted for different types of infectious diseases. Class A needs to be reported to the National Center for Disease Control and Prevention within 2 h of diagnosis, while class B and C need to be reported within 24 h of diagnosis [3]. When facing a multitude of diseases, making an accurate diagnosis of suspected infectious diseases is very important in the prevention and control of infectious diseases.

At present, there are few studies on the application of artificial intelligence (AI) methods for disease classification. Furthermore, existing research is mainly based on image data, such as X-rays, CT scans, MRIs, electrocardiograms (ECGs), and ultrasounds. Hannun et al. [4] used a deep neural network to detect and classify cardiac expert arrhythmias in a Holter monitor. Their results show good classification accuracy (area under curve = 0.97). Attia et al. [5] found the observed accuracy of an AI application on ECGs to be 85.7%. Wildman Tobriner et al. [6] showed that an AI-optimized thyroid imaging report and data system (TI-RADS) can moderately improve specificity and sensitivity compared to TI-RADS. Li Yang et al. [7] applied a neural network for the diagnosis of femoral head necrosis based on X-rays. They diagnosed femoral head necrosis based on the angle changes of the neural network learning image characteristics and recommended stages. S Sathitratanacheewin et al. [8] designed a DCNN to monitor lung nodules based on X-rays taken from the data of the National Institute of Health Clinical Centers and the National Library of Medicine Shenzhen No.3 Hospital. AI techniques are used in the detection of lymph node metastasis in women with breast cancer [9], skin cancer dermatological level classification [10], diabetic retinopathy and diabetic macular edema [11], and multiple diagnoses of Alzheimer’s disease [12]. However, there are few studies on using AI techniques to aid the decision-making applicable to infectious diseases. Rogachev et al. [13] used decision trees and Bayesian methods to classify and diagnose respiratory infections, where the final classification accuracy was 63.38%–70.68%. For COVID-19, Govindaraj et al. [14] used convolutional neural networks for feature extraction and classification based on chest CT image data and tried to achieve accuracy rate more than 90% of the COVID-19 classification model. Rajpurkar P et al. [15] considered the X-ray information of AIDS patients, using deep learning to help improve the diagnosis rate of tuberculosis in AIDS patients, with an accuracy rate of 79%. The only data used in this study were the original X-ray images; it lacked important textual information such as medical records. Although some studies explored the decision support of infectious disease diagnosis in the early stage, it is necessary to explore the research direction in combination with real text medical records. It is important to note that most current studies generally focus on a certain type of infectious disease based on image data. There are few effective methods for classifying a variety of infectious diseases simultaneously.

In this study, MIDDM, a model based on deep learning, is applied to support decision-making for infectious disease diagnosis recommendations. The infectious diseases required by the national health department to be reported [2] were selected for multi-classification clinical decision-making. The data source was clinical real data, covering 20,620 medical records from 2012 to 2020. The accuracy of the model is compared with common models such as Extreme Gradient Boosting (XGBoost), Bayesian model, Decision tree, and Logistic regression to estimate the prediction accuracy. This study first introduces the data used and the corresponding processing methods, then describes the currently popular models and introduces the research and the MIDDM structure. Next, the experimental results of the model are displayed and analyzed. Finally, we discuss the advantages, characteristics, and shortcomings of this study.

## Materials and methods

### Study design

**Fig. 1:**
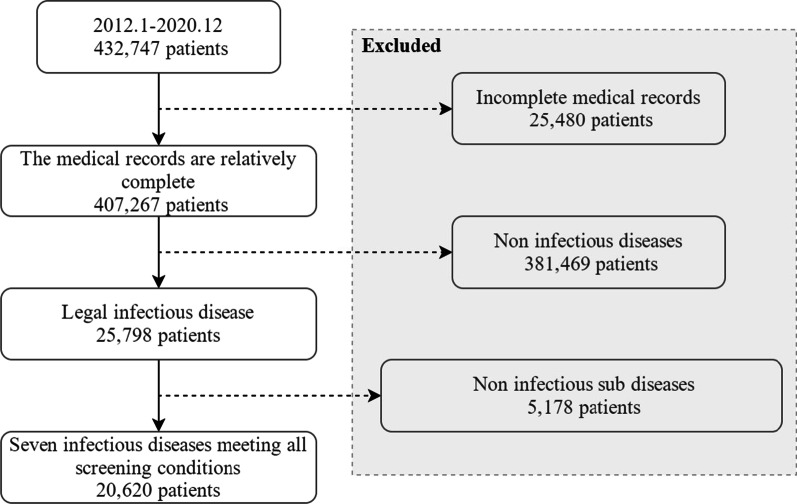
Flowchart of enrolment

In this study, patients admitted from 2012 to 2020 at a large general hospital were selected as the research object. First, we applied a quality control process to review the qualification of EHRs. Medical records with incomplete entries, inconsistent information, or follow-up medical records were discarded; 407,267 medical records remained. The dataset was then filtered according to the following inclusion criteria, as shown in Fig. 1: (1) The admission department must be the infection-related department. (2) Data related to non-communicable diseases is filtered out. (3) Other infectious diseases and non-infectious infectious sub diseases are filtered out. After screening, 20,620 medical records met the criteria, with the average age being 43.52 years old. 47.95% were men and 52.05% were women.

### Data

The diagnosis of infectious diseases requires patient medical records containing a variety of detailed information. In this study, the medical records were mainly obtained from the Medical Data Center of Peking University Third Hospital. Owing to the paucity of patients with infectious diseases, data from the outpatient and emergency department and the inpatient department are combined to expand the dataset. Data is extracted from unstructured electronic medical records (EMRs). As this data lacks uniformity, it should be processed in multiple steps before training the diagnosis model. For example, the alias and subclass names of the features and diseases used in the data are replaced by the Knowledge Base. We use regular expressions and natural language processing (NLP) methods to generate features. Then the training data are structured and vectorized. A wide variety of information that has an important impact on infectious diseases is considered. Table 1 presents the data used in the training model. Medicine diagnosis is used as the label of the sample, and the remaining extracted data is used as the input feature of the model. The data includes five kinds of documents, i.e., patient personal information, outpatient records, admission records, laboratory test reports, and examination reports.

**Table 1:** Key information extracted from medical records

Target information class	Specific extraction
Patient information	Age, gender, visiting time
Physical examination	Temperature, blood pressure, pulse, respiratory rate
Symptom	Diagnosis, symptom
Medical history	Main complaint, history of present illness, anamnesis, medication
Medical laboratory examination	Name of item, results
Examination reports	Name of examination item, results, value range

### Unstructured data processing

**Fig. 2:**
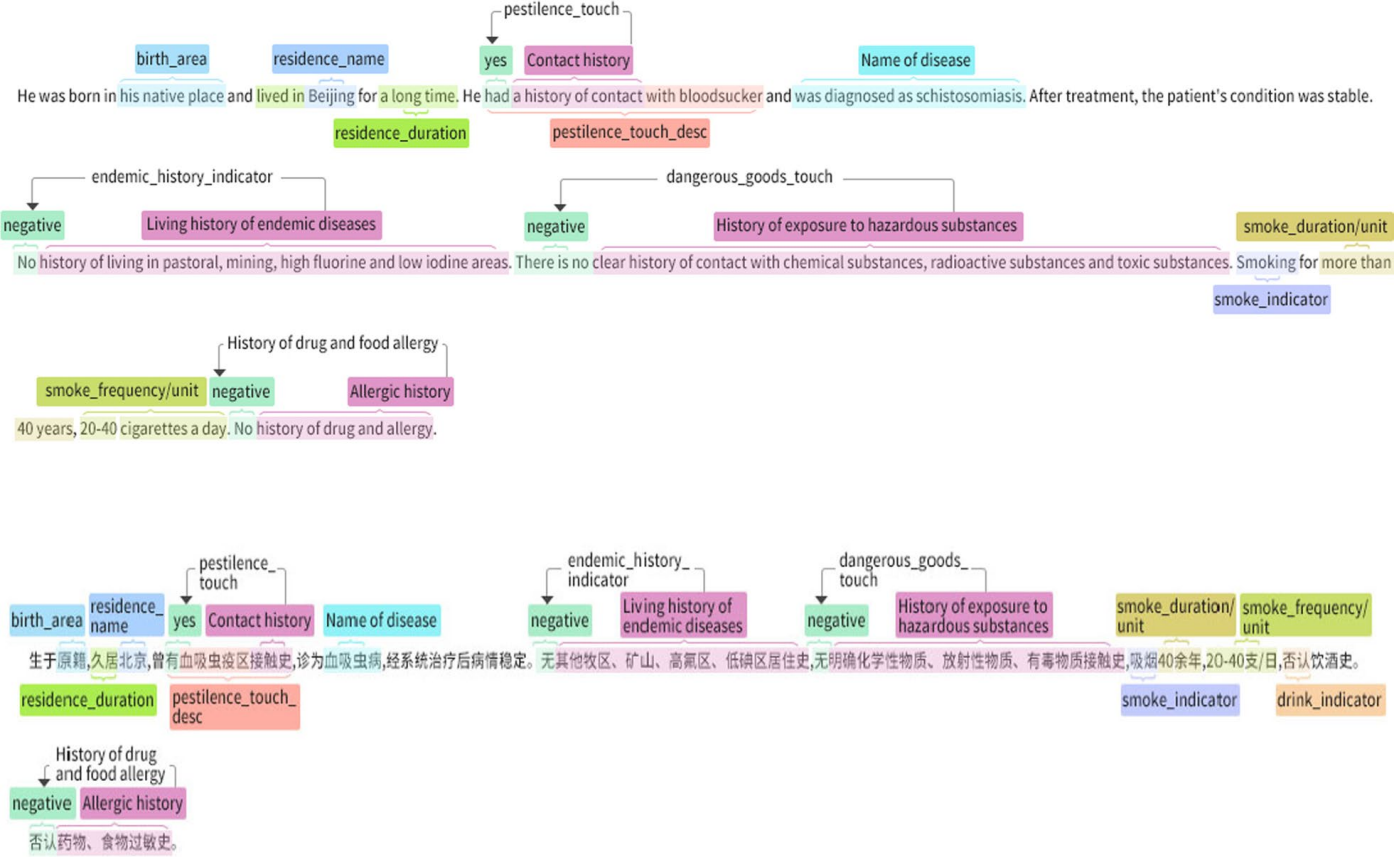
Sequence labeling of current case history based on BiLSTM-CRF model (English and Chinese)

The EMRs contains comprehensive, detailed, and accurate personal health information of patients. We deeply analyze and mine the information in the EMRs to obtain a large amount of potential information. However, in addition to the structured data such as medical laboratory results, unstructured free text data accounts for a large proportion of data in the EMRs. There are various ambiguities and potential polysemy in the free text in all areas. Model training usually finds it difficult to understand and use unstructured data. NLP can effectively transform these data into structured data that can be recognized by the model, which is the basis for constructing the model of infectious disease auxiliary diagnosis [16]. Sequence labeling is one of the core tasks in NLP for extracting information and mining deep semantics, including word tagging, named entity recognition, keyword extraction, and word meaning. The sequence annotation of EMRs can extract entities including diseases, symptoms, drugs, laboratory examinations, and the relationship between entities. This study is based on the current open-source method BiLSTM-CRF network [17], which performs sequence annotation well. Combined with the rule model and other methods, we use BiLSTM-CRF to realize the information extraction of the original EMRs. First, we input the serialized text after performing word segmentation into the BiLSTM layer, after which the forward and backward hidden state results are combined to generate the output of BiLSTM [18, 19]. Then, the output of BiLSTM is sent to CRF as the input, forming a BiLSTM CRF network structure [20]. This structure combines the advantages of BiLSTM and CRF, based on the bidirectional LSTM component so that it can effectively keep the information before and after the whole sentence [21] and extract the feature information in the sentence. With the help of the CRF layer, it can effectively learn the constraint information in the learning corpus and improve the accuracy of information extraction, as shown in Fig. 2.

Next, the extraction results of BiLSTM and CRF are stored in the database. At this time, the patient's medical record data is transformed from free text into structured field feature data. To use structured data to train models, we also need to process them via feature engineering, so that the field data can be input into the deep learning model. The discrete and continuous numerical features in the data are processed. For continuous numerical features, such as body temperature, diastolic blood pressure, and systolic blood pressure, abnormal values shall be processed first, and those significantly deviating from the normal value range shall be filtered out. Then, in order to eliminate the adverse effects of different dimensions between different features on model training, the continuous features will be normalized to the range [0,1]. For discrete features, such as gender, symptoms, and past diseases, the name is first standardized, and entity aliases such as symptoms, diseases, and signs are then replaced with standard names. In addition, the feature name and its chapter name shall be spliced. Different chapters in the medical record may contain the same entity information, but they have different medical meanings in medicine. For example, the name of the symptoms in the main complaint is the same as that at the time node in the current medical history, but one represents the current main symptoms while the other represents the symptoms that have appeared before, i.e., the past history. Therefore, we need to splice feature names based on chapters, such as "main complaint"_ "Femoral neck fracture" and "previous history"_ Femoral neck fracture ". Finally, one-hot coding is conducted to represent the original category features with 0/1 in high-dimensional space. Table 2 presents the data after word segmentation converts it into a feature; a value of 1 indicates that it has this feature, while 0 indicates that it does not have this feature.

**Table 2:** Data transformed into features after NLP word segmentation

Case number	Main complaint_ Femoral neck fracture pain	Past history_ Femoral neck fracture	Main complaint_ Symptoms_ Chest pain	Main complaint_ Symptoms_ fever	Temperature
1	1	0	1	1	0.91
2	0	1	0	1	0.89

After processing continuous and discrete features, 395,950 dimensional features are obtained as model input data, including patient personal information having 2-dimensional characteristics (gender and age). The outpatient records and admission records contain 354,589 dimensional characteristics. The laboratory test reports contain 1742 dimensional characteristics. Examination reports contain 39,619 dimensional features. In addition, the training label of the sample is obtained by diagnosis through one-hot coding. After the above processes, a total of 20,620 samples were obtained. The number of samples of each infectious disease category is listed in Table 3.

**Table 3:** Number of samples of each infectious disease category

Infectious disease category	Viral hepatitis	Influenza	Hand foot and mouth disease	Tuberculosis	Syphilis	Infectious diarrhea	Measles
Number of samples	3663	5007	3616	5834	1500	730	270

It can be seen from Table 3 that there is an imbalance in the number of samples in this multi-category data. In order to alleviate the impact of data imbalance on the model results, this study adopts multiple sampling of a few samples and category weight measures. Among them, multiple sampling involves random sampling 1.5 times and random sampling 2 times for measles and infectious diarrhea, respectively. After sampling, the number of infectious diarrhea samples increased to 1095 and the number of measles samples increased to 540.

Category weight is added to make the category with fewer samples have higher calculation weight and get more learning in model training. $w_{k}$ represents the weight of class k, $N_{all}$ represents the total number of samples in the dataset, C represents the total number of categories (C = 7 in this experiment), and $N_{k}$ represents the number of samples in category K. When the weight is not changed, the weight of each category is $\frac{1}{C}$ of the average attention. We assume that the calculation formula of the weight satisfies:

Category weight × Proportion of category samples in the total dataset = Average attention

1 $$w_{k} = \frac{{N_{all} }}{{C \cdot N_{k} }}$$

### Model

The classification machine learning method is usually used for the diagnosis of infectious diseases. Under the current multi-classification task of simultaneous diagnosis of multiple infectious diseases, we also considered using the classification machine learning method. With a two-class machine learning model, we use a multi-classification strategy (such as One-VS-Rest strategy) to transform it into a multi-class architecture.

#### Logistic regression model

Logistic regression model is a binary classification algorithm based on the combination of linear regression model and sigmoid activation function [22]. The model has a simple structure. Compared with the deep neural network, the logistic regression model only has a single-layer weight, so its weight can be understood well [23]. The value range of the model output is within [0, 1], which can be regarded as the probability of belonging to a certain class. In the infectious disease diagnosis task for this research, we use strategies such as One-VS-One or One-VS-Rest to transform the binary classification model into a multi-class prediction architecture.

#### Multiple infectious disease diagnostic model (MIDDM)

**Fig. 3:**
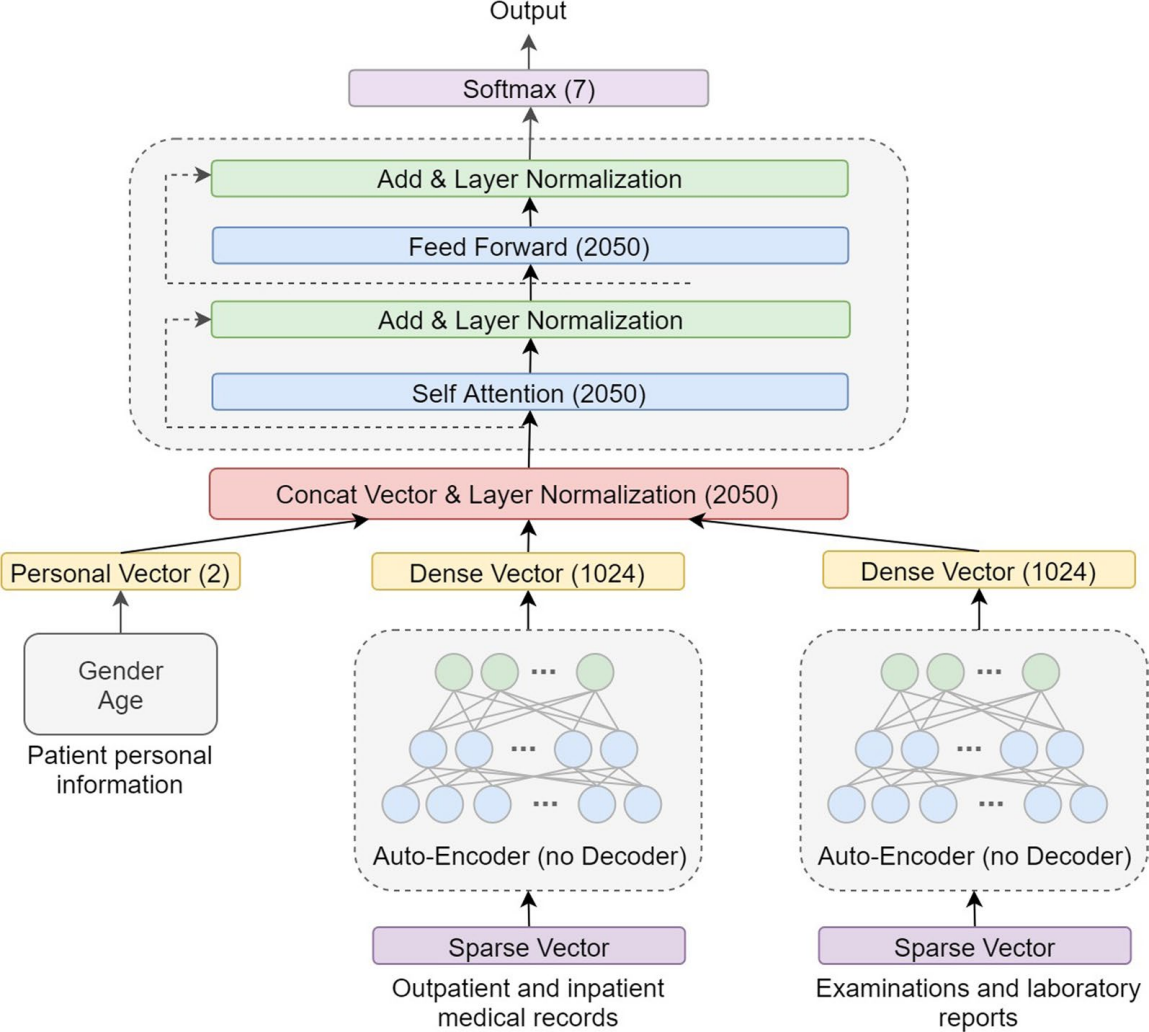
Multiple infectious disease diagnostic model structure

**Fig. 4:**
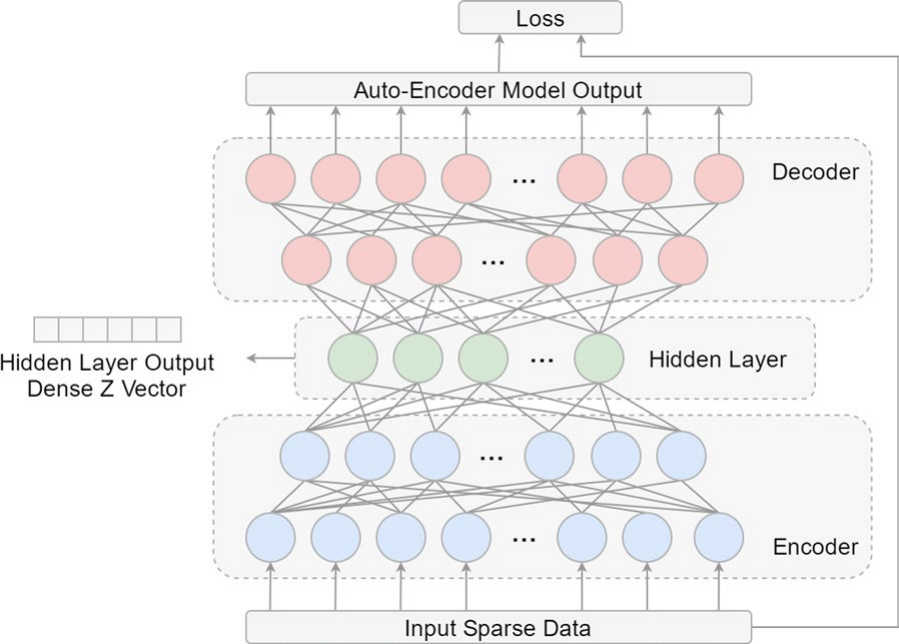
Auto-encoder model structure and hidden layer output Z vector

The basic structure of the multi-class neural network has an input layer, hidden layer, and output layer [24]. When the neural network is applied to multi-classification tasks, the softmax function should be used as the activation function in the final output layer, so that the model can calculate the classification probabilities of multiple categories simultaneously, with the category with the highest probability being the final diagnosis output [25]. In this study, a multiple infectious disease diagnostic model (MIDDM) was constructed for a variety of common infectious diseases. Figure 3 shows the structure of the MIDDM. Owing to high-dimensional sparse data (that is, data with more 0 values), the computational complexity in training is relatively high and the model is difficult to optimize [26]. Therefore, it is necessary to use the method to compress the data and extract the features. Given the large amount of sparse data in medical data, MIDDM introduces the auto-encoder deep learning model [27] and uses unsupervised learning [28, 29] to perform efficient feature extraction and feature representation on high-dimensional data. The auto-encoder can be used to densify sparse data so that the model is easier to train and achieves better results. In the optimization process, the Auto-Encoder does not need to use the infectious disease category to which the sample belongs as the label, but learns the characteristics of the sample as the input of the neural network and the label of the model concurrently. By minimizing the reconstruction error, it learns the abstract characteristics of the sample to represent the Z vector (the output vector of the middle hidden layer). The structure of the Auto-Encoder model applied in this study is shown in Fig. 4, which mainly includes encoder, decoder, and hidden layer. Encoder and decoder both contain two-layer neural networks. The number of neurons in the two-layer network of encoder ranges from more to less. By contrast, the hidden layer in the middle has only a single neural network. The auto-encoder first compresses the original high-dimensional sparse vector to the low-dimensional hidden layer through the encoder neural network, and then restores the output of the hidden layer to the original feature dimension through the decoder. The smaller the loss between the final model output and the original feature calculation, indicating that the smaller the information lost in the process of compressing to the hidden layer, the more accurately the hidden layer can represent the original feature. After the pre-training of the auto-encoder, the decoder part in the model is deleted, and the Z vector output from the hidden layer is directly used as the dense representation of the original features and input into the subsequent classification model. Given that different medical records contain different types and numbers of features, we construct different auto-encoder models for different records in the process of densification, so as to obtain their own more effective abstract feature expressions. Specifically, the number of neurons in the two layers of the encoder is 4096 and 2048 respectively; the number of neurons in the hidden layer is 1024; the number of neurons in the two layers of the decoder is 2048 and 4096 respectively, and finally the 4096 dimension of the decoder output is mapped back to the input feature dimension and the input data to calculate the loss. According to the model structure, two auto-encoders are trained, one each for outpatient data/admission record and inspection report. Finally, each auto-encoder takes the 1024 hidden layer output as the dense vector representation of the original high-dimensional sparse data and inputs it into the subsequent self-attention module.

Next, the decoder part of the auto-encoder is deleted after training, and the remaining structure is combined with the self-attention module. Specifically, the dense data output by the hidden layer in the two auto-encoders are combined with the coding vector containing the patient's personal information, with a total of 2050 dimensional features. Before being input to the subsequent classification structure, the merged vectors are normalized by the layer normalization method. Finally, the results are input into the self-attention module. The dense vectors obtained from different documents after different auto-encoders do not belong to the same feature value space. Layer normalization is used to normalize the entire vector to reduce the impact of the above-mentioned issues on the training results. Self-attention and residual learning are mainly introduced in the calculation structure of MIDDM. The dense vector and result after self-attention calculation are added and then the result of the addition and the result after the feed-forward calculation are directly summed. The model finally uses softmax as the activation function to output the respective probabilities of multiple infectious diseases, so as to complete the simultaneous classification of multiple infectious diseases.

### Effect evaluation

The MIDDM model predicts that the first diagnosis is correct if it is consistent with the patient’s clinical diagnosis; otherwise, it is incorrect. According to medical safety management requirements, infectious diseases are different from other non-communicable diseases and are managed separately. Therefore, the diagnostic accuracy of infectious diseases does not consider the order of diagnosis.

The confusion matrix predicted by the multi-classification model is presented in Table 4 (three categories are listed as examples).

**Table 4:** Confusion matrix predicted by the multi-classification model

Real label	Prediction results		
	Class 1	Class 2	Class 3
Class 1	$TP_{1}$	$E_{1,2}$	$E_{1,3}$
Class 2	$E_{2,1}$	$TP_{2}$	$E_{2,3}$
Class 3	$E_{3,1}$	$E_{3,2}$	$TP_{3}$

$TP_{k}$ represents the number of samples whose real label is k for which the model predicts k; $E_{k,i}$ represents the number of samples whose real label is k for which the model predicts i; C represents the total number of categories of multiple classifications. According to the definition of the multi-classification confusion matrix, the overall prediction performance and accuracy of the model are evaluated. The specific formula is:

2 $$Accuracy{ = }\frac{{\sum\nolimits_{k}^{C} {TP_{k} } }}{{\sum\nolimits_{k}^{C} {TP_{k} + \sum\nolimits_{k}^{C} {\sum\nolimits_{i \ne k}^{C} {E_{k,i} } } } }} \times 100\%$$

In order to more comprehensively improve the prediction performance of the multi-classification model, precision and recall are used for evaluation.

3 $$\Pr ecision_{k} { = }\frac{{TP_{k} }}{{TP_{k} + \sum\nolimits_{i \ne k}^{C} {E_{i,k} } }}$$

4 $${\text{Re}} call_{k} { = }\frac{{TP_{k} }}{{TP_{k} + \sum\nolimits_{i \ne k}^{C} {E_{k,i} } }}$$

## Results

This section introduces the results of the classification study using the MIDDM for the diagnosis of multiple infectious diseases. All data used in the experiment comes from the actual medical records of hospitals. First, the infectious disease data is used to filter the normalized infectious disease names and then screen out the sub-diseases that are not infectious under the sub-categories of tuberculosis. For example, thyroid tuberculosis and renal tuberculosis, which appear under the sub-category of tuberculosis, are not infectious. Next, in order to ensure the balance of the data used in the training model and the testability of the model’s predictive ability, infectious diseases with fewer than 10 cases per quarter were eliminated. Finally, seven infectious diseases were predicted and verified. In order to make the input data for the MIDDM for training, we use the word segmentation and entity recognition method realized using NLP technology to extract the features of infectious disease records and transform the format through One-Hot Encoding. Finally, we obtain 20,620 samples of high-quality medical records, which can be used for research. Training data, consisting of 16,496 samples, accounts for 80%, while test data, consisting of 4124 samples, accounts for 20%. MIDDM compresses the 395,936-dimensional sparse data into a 1024-dimensional dense vector through the auto-encoder with 1024 neurons in the abstraction layer. The number of feed-forward neurons in the classification structure is 256. In this study, 32 epochs were trained on the model with a learning rate of 0.001. In the process, only the model with the smallest loss of the test set is retained, and the training is stopped when the loss exceeds 10 epochs. The number of effective training epochs is 32. In addition, we also compared auto-encoders with different numbers of hidden layer neurons, which are 256, 512, 1024, 2048, and 4096 respectively. The results in Table 5 represent that the number of hidden-layer neurons increases from 256 to 1024, and the subsequent multi-classification results are improved. However, when the number of neurons is more than 1024, the accuracy of the model does not improve noticeably. Considering the model size, calculation efficiency, and subsequent practical deployment and application, 1024 is finally selected as the optimal number of hidden layer neurons.

**Table 5:** Final model prediction results after auto-encoder pre-training with various numbers of neurons

Number of neurons	256	512	1024	2048	4096
Test set accuracy	82.71%	86.03%	89.52%	89.74%	89.67%

Via the experiment, it is found that because the goal of the model is to solve the multi-classification task of predicting all categories simultaneously, it is equivalent to training the same number of epochs for the prediction of all categories, which is more vulnerable to the unbalanced number of category samples. In order to alleviate the problem of unbalanced samples, the measures of category weight and multiple sampling for categories having a small number of samples are added in this study. Finally, the overall prediction accuracy of all infectious diseases in the test set is 89.52%. The respective results of each infectious disease are presented in Table 6.

**Table 6:** Training and test results for MIDDM

Infectious disease	Number of training samples	Training accuracy (%)	Number of test samples	Testing recall (%)	Testing precision (%)	F1-score
Viral hepatitis	2954	99.86	709	99.44	87.04	0.8704
Influenza	3924	98.47	1083	95.38	91.42	0.9142
Hand foot and mouth disease	3015	97.31	601	95.17	88.82	0.8882
Tuberculosis	4630	95.01	1204	86.88	94.66	0.9466
Syphilis	1208	83.03	292	72.60	89.45	0.8945
Infectious diarrhea	575	87.30	155	60.65	72.31	0.7231
Measles	190	42.11	80	37.50	44.12	0.4412

**Fig. 5:**
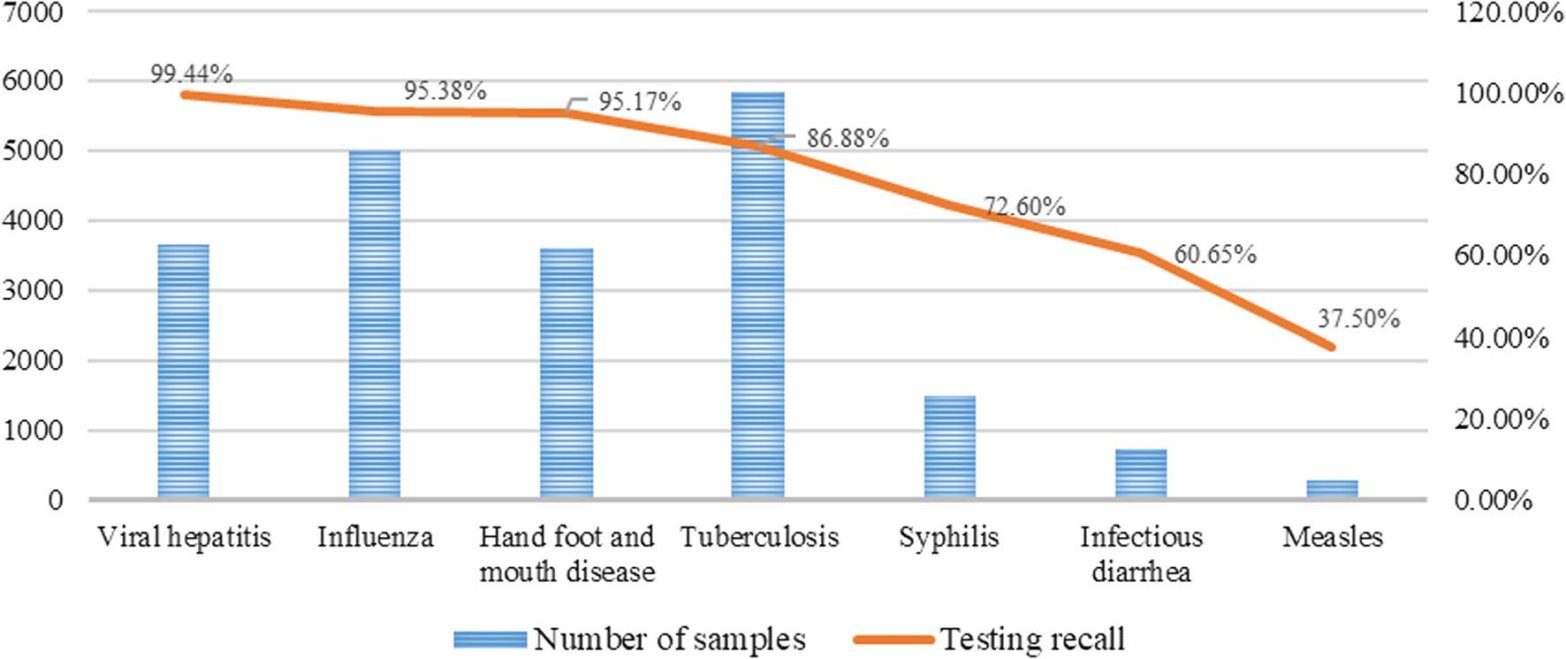
Relationship between the diagnostic recall of infectious diseases and the number of samples

It can be seen from Table 6 that MIDDM has achieved better prediction results in the diagnosis experiment for various kinds of infectious diseases. In the case of similar diseases, characteristics used for diagnosis are similar and the category weight is adjusted. The prediction accuracy of disease classification with more sample data is significantly better than the prediction accuracy of disease classification with fewer sample data. For example, the training data for viral hepatitis, influenza, and hand foot and mouth disease were 2954, 3924, and 3015 cases respectively, and the corresponding test recall rates were 99.44%, 95.38%, and 95.17%. By contrast, syphilis, infectious diarrhea, and measles have less training data, i.e., 1,208, 575, and 190, and the corresponding test recall rates are 72.60%, 60.65%, and 37.50%, respectively. The increase of the interference factors of the disease diagnosis feature also directly affects the prediction accuracy rate. For example, the sample size of tuberculosis is 4630 and the prediction accuracy rate is 86.88%. For the classification results of tuberculosis, although the sample size is up to 4630, the result is not significantly better than that of viral hepatitis with the sample size of 2954. The main reason is that tuberculosis has similar symptoms to many other diseases, such as lung cancer, pneumonia, and chronic obstructive pulmonary disease. Furthermore, tuberculosis also involves multiple variations such as positive and negative etiology, and the clinical diagnosis of tuberculosis is also more complicated than that of viral hepatitis, influenza, and other diseases. However, for infectious diseases with a very small amount of data such as measles, the characteristics of infectious diseases cannot be fully learned during training and the accuracy of the training set is low; therefore, the accuracy of the test set is also low and the model cannot be widely verified using a small test set. For diseases with fewer data samples, the amount of data needs to be increased to further prove the effectiveness of the model. Figure 5 shows the recall of the diagnosis and classification of the corresponding model for each infectious disease when there are different numbers of training samples.

## Discussion

### Performance of different NLP models

In information extraction, we compare the traditional machine learning models CRF, HMM and deep learning models LSTM-CRF and BiLSTM-CRF. The model is applied to the identification of 20,620 electronic medical record data with five types of entity labels: disease diagnosis, symptom, medicine, laboratory test, and imaging examination. Training data, consisting of 16,496 samples, accounts for 80%, while test data, consisting of 4124 samples, accounts for 20%. After training, the comparison conclusion is presented in Tables 7 and 8.

**Table 7:** Recognition accuracy and recall rate of five types of entities (%)

Model	Disease diagnosis		Symptom		Medicine		Laboratory test		Imaging examination	
	Accuracy	Recall	Accuracy	Recall	Accuracy	Recall	Accuracy	Recall	Accuracy	Recall
HMM	71.4	78.0	77.9	84.5	69.8	72.6	86.3	88.7	80.6	88.2
CRF++	69.7	79.2	78.1	80.5	77.2	84.6	89.6	90.8	80.2	78.8
LSTM-CRF	85.3	87.5	81.8	**87.8**	82.5	**91.2**	90.2	91.5	89.6	88.5
BiLSTM-CRF	**88.4**	**90.1**	**87.5**	**87.8**	**91.8**	90.6	**91.2**	**92.6**	**95.3**	**94.1**

CRF++ is an open source implementation tool for CRF. It is essentially a CRF algorithm. It is the CRF tool with the best comprehensive performance at present

**Table 8:** F1-socre of five types of entity recognition (%)

Model	Disease diagnosis	Symptom	Medicine	Laboratory test	Imaging examination	Average
	F1-score	F1-score	F1-score	F1-score	F1-score	F1-score
HMM	74.6	81.1	71.2	87.5	84.2	79.7
CRF + +	74.1	79.3	80.7	90.2	79.5	80.5
LSTM-CRF	86.4	84.7	86.6	90.8	89.0	87.5
BiLSTM-CRF	**89.2**	**87.6**	**91.2**	**91.9**	**94.7**	**90.9**

Overall, the deep learning model performs better than the traditional machine learning model. The F1-score of BiLSTM-CRF model is 90.9% on average in five types of entities, which is better than 87.5% of LSTM-CRF model, especially in imaging examination entity. Thus, it can be seen that the two-way LSTM structure better identifies the entity boundary.

### Performance of different models

**Fig. 6:**
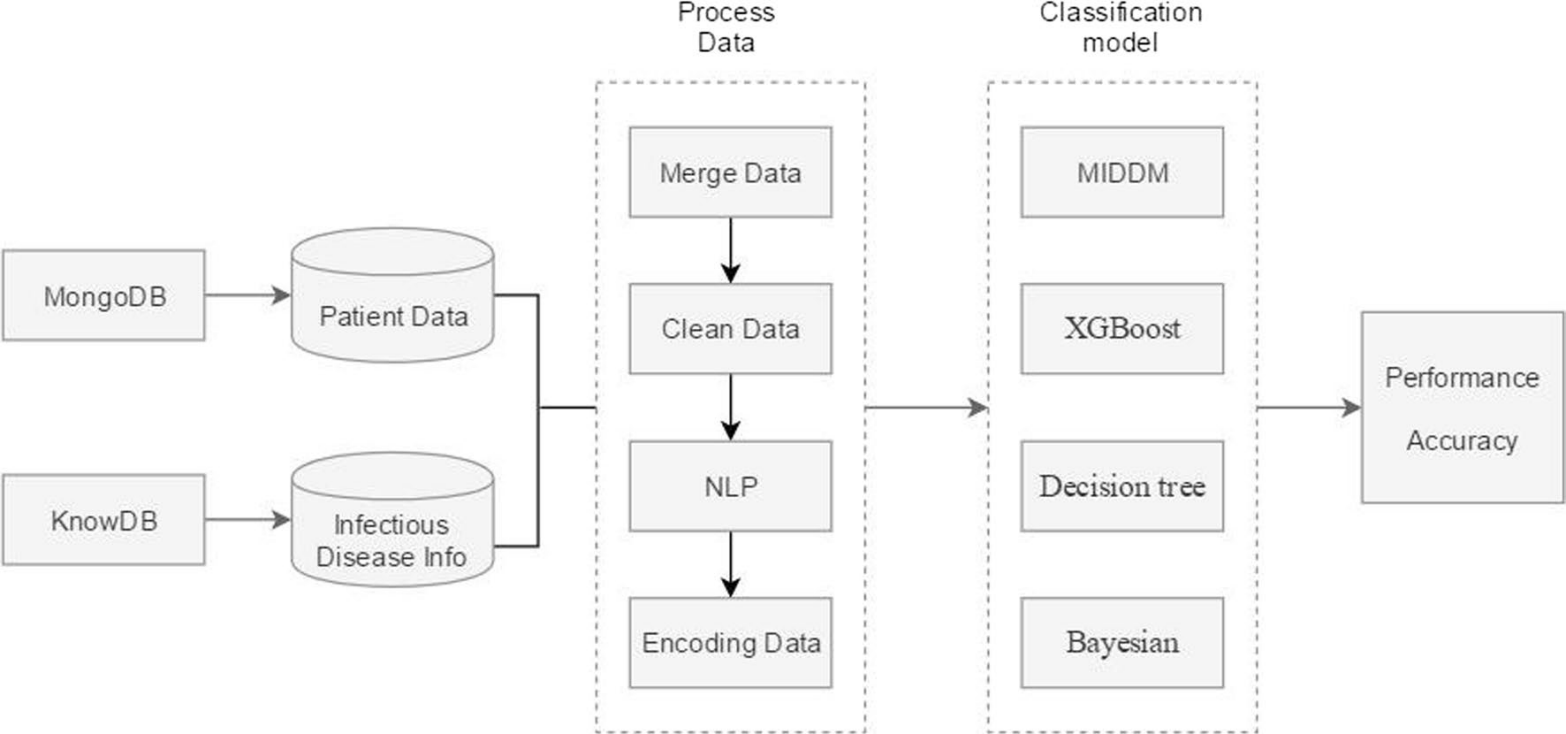
Work flow

MIDDM model is compared with other models used in other studies. The process is shown in Fig. 6. With the same data source being used, the comparison conclusion is presented in Table 9. The MIDDM model is superior to other models in terms of the multi-classification of infectious diseases. Even for tuberculosis, which is difficult to partition, the MIDDM model is also significantly better than other methods. This indicates the superiority of the model in the multi-classification of unstructured medical records of infectious diseases.

**Table 9:** Comparison of the accuracy of infectious disease diagnosis between MIDDM and other models

Infectious disease	MIDDM (%)	XGBoost (%)	Decision tree (%)	Bayesian (%)	Logistic regression (%)
Viral hepatitis	99.44	96.19	90.13	85.19	91.26
Influenza	95.38	91.51	89.47	82.27	90.49
Hand foot and mouth disease	95.17	90.03	88.29	84.44	85.49
Tuberculosis	86.88	83.08	80.21	76.29	82.31
Syphilis	72.60	70.75	70.28	65.09	68.87
Infectious diarrhea	60.65	56.38	56.38	54.26	56.38
Measles	37.50	36.25	32.50	33.75	35.00

This study also analyzes the reasons for the performance of different models. The Bayesian model is a statistical model that predicts by calculating feature and label conditional probabilities. The calculation theory of the model is simple but its simplicity also restricts its performance in classification tasks. We set the Laplacian Smoothing to 1e−9. In addition, the application of the Bayesian model must meet the assumption of conditional independence among various features and the high-dimensional medical records data used in the current research cannot meet the above assumptions. Therefore, it is difficult for the Bayesian model to have better classification results than MIDDM. A decision tree is a common classic machine learning model. It has strong interpretability and can process data with missing features. However, this model is prone to overfitting. Furthermore, owing to attribute division based on information gain, different judgment criteria will produce different attribute selection tendencies. The decision tree model used for comparison uses the Gini coefficient [30] as the node judgment algorithm. In order to prevent overfitting, the maximum depth is 5 and the minimum number of leaf node samples is 2. In the research of multi-classification of infectious diseases, there are differences in the amount of data of each category and the number of attribute features, which does not allow the decision tree model full play to its advantages when working on the current data. XGBoost is an improved model with better prediction performance and has been more widely used in recent years. The model uses a tree model as a base model, simultaneously applies the first derivative and the second derivative, and approximates the training model by learning residuals. We set the maximum depth of the XGBoost tree as 6, learning rate as 0.05, alpha value as 0.01, and gamma value as 0.05 in the experiment. Although the XGBoost model can achieve better classification performance [31], it finds it difficult to achieve fast model iteration and optimization. In order to deal with the high-dimensional sparse medical data used in current multi-classification tasks, this study proposes the MIDDM deep learning model. This model can construct different auto-encoders for different document data. It also performs abstract and dense representation of high-dimensional sparse features while independently retaining the original feature information of each document. Following that, self-attention, residual learning, and feed-forward neural network constitute the core structure of classification. Finally, the softmax layer is used to weight the multi-classification results. This model alleviates the adverse effects of high-dimensional sparse data and has strong generalization capabilities while having excellent fitting capabilities.

### Practical significance of MIDDM

Before having the MIDDM model to assist in the diagnosis of clinical infectious diseases, doctors needed to diagnose infectious diseases based on their experience. In actual medical scenarios, most infectious diseases do not have designated clinics and are companion diagnoses of other diseases. Through MIDDM, doctors can be notified as soon as possible and the patient can be contacted for diagnosis or follow-up diagnosis of infectious diseases, so as to prevent the spread of infectious diseases in society.

### Limitations

In hospitals, some infectious diseases are rare, such as cholera and plague. For these rare diseases, it is difficult to learn from the existing data of the hospital, so we use standard diagnosis and treatment guidelines for infectious diseases classification [32]. When the medical record content triggers the rule, it can be reminded of infectious diseases. This study also has some limitations. First, owing to the quality of medical records and other factors, the amount of infectious disease data used in this study is small, accounting for only 10.1%. Second, this study did not consider the national adjustments to the diagnostic criteria for infectious diseases from 2016 to 2021, which prompted a significant difference between physicians’ initial medical records and the lab reports, which led to the accuracy of the model input features being unstable. For example, in order to facilitate monitoring, changes were made to the medical history collection of influenza after COVID-19, resulting in changes in model input features. Third, this research is mainly aimed at one hospital and its collaborative institutions in Beijing, China. The incidence of some infectious diseases is low and the data samples are not enough to support MIDDM training and verification. Therefore, for infectious diseases such as brucellosis and echinococcosis that have an extremely low incidence, there may be a certain gap between the coverage of infectious diseases within the scope of our study and within other regions in China.

## Conclusions

This research is based on the real infectious medical records of the hospital and establishes a basic dataset through data collection throughout the course of various diseases. Using the unsupervised learning method of an auto-encoder model to extract and express the features of high-dimensional data efficiently, and dense the sparse data, so that the model is easier to train. In order to improve the performance of the MIDDM deep learning model, residual network and attention mechanism are introduced. MIDDM has achieved better prediction results in the diagnosis experiment for several kinds of infectious diseases. In the case of similar disease diagnosis characteristics and similar interference factors, the prediction accuracy of disease classification with more sample data is significantly better than the prediction accuracy of disease classification with fewer sample data. This study proposes experiments with the MIDDM model and other models used in other studies. Taking viral hepatitis as an example, the accuracy of MIDDM is 99.44%, which is significantly higher than that of XGBoost (96.19%), decision tree (90.13%), Bayesian method (85.19%), and logistic regression (91.26%). This is true for other diseases as well. These findings confirm the role of AI-based assisted decision-making for diagnosing infectious diseases with improved diagnosis efficiency. It is of considerable significance for early screening and early warning of infectious diseases. Infectious diseases are more sensitive than other non-communicable diseases and need to be diagnosed with higher accuracy. Therefore, in the future, it is necessary to combine the group experiment, carry out retrospective research, and create early diagnosis plans for uncommon infectious diseases.

## Data Availability

The data that support the findings of this study are available from the corresponding author upon reasonable request.

## Abbreviations

MIDDM: Multiple infectious disease diagnostic model
NLP: Natural language processing
EMR: Electronic medical record
